# Complications of Laser and Energy‐Based Procedures in Dermatology: Classification, Management, and Prevention

**DOI:** 10.1111/ddg.70102

**Published:** 2026-03-09

**Authors:** Lynhda Nguyen, Wolfgang Kimmig, Stefan Hammes, Stefan W. Schneider, Nikolaus Seeber

**Affiliations:** ^1^ Department of Dermatology and Venereology University Medical‐Center Hamburg‐Eppendorf Hamburg Germany; ^2^ Department for Oral and Maxillofacial Surgery/Plastic Surgery University of Greifswald Greifswald Germany; ^3^ Dermatology Practice Dres. Peter/ Seeber/ Altheide/ von Georg Hamburg Germany

**Keywords:** adverse events, complications, energy‐based devices, hyperpigmentation, hypopigmentation, laser, patient safety, scarring

## Abstract

Laser and energy‐based devices (EBDs) are firmly established in dermatology and widely used for both medical and aesthetic purposes. Even with advanced safety features such as integrated cooling and real‐time monitoring, these procedures still carry inherent risks. Complications may occur as a result of inappropriate device choice, suboptimal parameter settings, insufficient patient evaluation, or limited operator experience. This CME article provides an overview of the spectrum of adverse events associated with dermatologic laser and EBD treatments, ranging from expected short‐term reactions to late complications, including pigmentary alterations, scarring, and ocular injury. Strategies for prevention and management are outlined, with particular attention to the importance of patient selection, treatment planning, and post‐procedure care. In addition, regulatory frameworks in Germany, such as the *Verordnung zum Schutz vor schädlichen Wirkungen nichtionisierender Strahlung bei der Anwendung am Menschen (NiSV)*, and initiatives like the complications registry of the German Dermatological Society of Laser Medicine are highlighted as important measures to enhance patient safety. A comprehensive understanding of potential risks and their mitigation is essential to ensure safe practice and optimize clinical outcomes in laser dermatology.

## INTRODUCTION

The integration of laser and energy‐based devices (EBDs) into dermatologic practice and other medical disciplines has profoundly transformed the management of both medical and aesthetic concerns. Over the past decades, the therapeutic armamentarium has expanded from traditional surgical and pharmacologic interventions to include a wide range of devices such as pulsed‐dye lasers, ablative and non‐ablative fractional lasers, nanosecond and picosecond lasers, intense pulsed light, radiofrequency, and other systems.^1^, ^2^ These modalities have not only broadened treatment possibilities but also raised patient expectations for minimally invasive procedures with reduced downtime and improved cosmetic outcomes.

A cornerstone of modern laser dermatology is the principle of selective photothermolysis, first described by Anderson and Parrish in 1983.^3^ This concept underpins the safety and precision of many laser treatments by enabling the selective destruction of targeted chromophores – such as hemoglobin, melanin, or water – while largely sparing the surrounding tissue from collateral damage.^3^ A key factor in achieving this selectivity is the thermal relaxation time (TRT) of the target structure, which represents the time required for the target to lose 50 % of its heat. To confine thermal injury effectively, the laser pulse duration must be equal to or shorter than the TRT of the chromophore.^3^, ^4^ Building on this principle, contemporary devices additionally incorporate advanced safety features. These include integrated cooling systems, as well as real‐time temperature and impedance monitoring, which provide dynamic feedback during treatment to reduce the risk of overheating or excessive tissue injury.^5^, ^6^, ^7^, ^8^

Despite these technological advancements, complications cannot be completely eliminated.^9^, ^10^ The occurrence and severity of side effects or complications depend on multiple variables, including the selection of device, treatment parameters, patient‐specific factors, and the expertise and experience of the treating physician. While some complications are transient and self‐limited, others can be more serious, with long‐term functional or aesthetic consequences. Against this backdrop, the present article synthesizes evidence from the literature alongside clinical experience to provide a discussion of the aetiology, classifications of complications of lasers and EBDs, and strategies for prevention.

## AETIOLOGY AND RSIK FACTORS

Complications in laser and EBD procedures arise from patient‐related, procedure‐related, and operator‐related factors. Careful consideration of each of these elements is essential to ensure safety and optimize treatment outcomes.

### Patient‐Related Factors

Among patient‐related factors, the Fitzpatrick skin type is the most critical.^11^ Individuals with skin of color are at greater risk of complications because increased melanin in the epidermis absorbs part of the laser energy intended for other chromophores such as water, hemoglobin, or other melanin‐containing structures. This competitive absorption reduces treatment selectivity and predisposes to pigmentary disturbances such as post‐inflammatory hyperpigmentation or hypopigmentation.^12^, ^13^

Comorbidities further shape risk profiles. Patients with photosensitivity disorders, or those taking photosensitizing medications such as tetracyclines or amiodarone, may develop exaggerated inflammatory reactions.^14^ For patients taking photosensitizing medications, it is advisable, when feasible, to perform test spots or to discontinue the medication, depending on the planned procedure. The 2009 guidelines of the British Medical Laser Association stated that non‐essential aesthetic laser treatments are contraindicated in patients taking drugs that cause either systemic or localized photosensitization.^15^ However, up to 2014, no published reports documented adverse effects of laser treatments in patients using photosensitizing medications.^16^ To our knowledge, no specific guidelines have since been established regarding recommended discontinuation periods or a standardized list of implicated drugs.

Similarly, the presence of active bacterial, viral, or fungal infections can lead to local spread or worsening of the condition if treated with lasers or EBDs. Herpes simplex reactivation is a well‐recognized complication, especially in ablative laser treatments.^17^, ^18^

Another crucial aspect is the role of ultraviolet exposure. Both recent and post‐procedure sun exposure increase the likelihood of pigmentary changes and burns.^19^ For this reason, comprehensive patient education before and after treatment is essential. Counseling should emphasize strict sun protection four weeks pre‐ and four weeks post‐treatment. The use of a sunscreen with at least SPF 50 is recommended, along with additional protection provided by appropriate clothing. If this cannot be reliably ensured, especially during the summer months, a pause in treatment should be considered. Decorative cosmetics on the area to be treated should be avoided or removed prior to treatment.

Equally important is guidance on post‐treatment wound care and infection prevention.^20^, ^21^ Such preventive measures not only improve safety but also align expectations with realistic outcomes and enhance patient satisfaction.

Among patient‐related risk factors, the Fitzpatrick skin type and ultraviolet exposure are the most critical.

### Procedure‐ and operator‐related factors

In addition to patient‐related aspects, procedure‐ and operator‐specific parameters play a central role and may be responsible for the majority of laser and EBD‐related complications.^22^ The choice of device and treatment settings must be carefully tailored to the indication and to the patient's skin type.

Using an inappropriate device or mismatched parameters substantially increases the risk of complications. High fluences, unsuitable pulse durations, or inadequate cooling can cause epidermal and dermal injury, including burns, scarring, and pigmentary changes. Ablative resurfacing procedures, while highly effective, inherently carry greater risks than non‐ablative methods, and their safety depends heavily on precise parameter selection and execution. Non‐ablative procedures generally offer a more favorable safety profile, particularly in skin of color, but even these can result in adverse events if performed without sufficient caution. Safe and effective use of lasers and EBDs requires a solid understanding of laser–tissue interactions as well as extensive practical experience. Insufficient training, limited exposure to a diverse patient population, or over‐reliance on manufacturer settings can lead to avoidable complications.

Competent practice requires careful patient selection, realistic counseling, precise intra‐procedural technique, and vigilance for early signs of tissue injury, such as inappropriate pain, excessive erythema, blistering, grey discoloration, or burns.

## ANTICIPATED PHYSIOLOGICAL RESPONSES

Laser‐based dermatologic procedures frequently elicit short‐term reactions as a direct consequence of targeted thermal, photomechanical, or photochemical interactions with tissue.

The most common immediate effects include erythema, edema, purpura, and crusts, which are generally benign and self‐limited. These responses are anticipated, typically mild and self‐limiting, and therefore should be regarded as expected side effects rather than true complications.

Erythema is the most consistent response following laser and EBD treatments. Its severity and duration depend on device parameters such as wavelength, fluence, pulse duration, and depth of penetration, as well as patient‐related factors such as baseline skin sensitivity.^23^ While erythema typically subsides within several hours to a few days, its intensity may be attenuated through the use of intraoperative cooling systems and appropriate post‐procedure care.^5^, ^23^

Edema commonly accompanies erythema and reflects an acute inflammatory response. It tends to be more pronounced in regions with loose connective tissue, such as the periorbital area, and may persist for several days.^23^ Supportive measures, including cold compresses, head elevation, and in some cases anti‐inflammatory agents like topical steroids for a few days post‐treatment, can help alleviate discomfort and accelerate resolution.

Purpura arises when vascular‐targeting lasers, particularly pulsed dye devices, induce focal capillary rupture. In the context of vascular lesion treatment, purpura often represents a desired clinical endpoint.^24^ These maculae usually resolve spontaneously within one to two weeks without residual effects.

Crust formation and punctiform bleeding may occur following ablative laser treatments.^25^ Crusts are usually transient, resolving within several days to weeks as re‐epithelialization proceeds.^25^ Adequate wound care, including gentle cleansing and emollient application, is critical to prevent secondary infection and support optimal healing.

Although these immediate reactions are transient and rarely clinically significant, patient counseling prior to treatment remains essential. Clear communication regarding expected short‐term changes, along with instructions for post‐treatment care, helps to manage expectations, reduce anxiety, and promote adherence to aftercare recommendations.

## IMMEDIATE COMPLICATIONS

Immediate complications following laser and EBD procedures typically occur within hours to days after treatment. While most are transient and self‐limiting, some may require medical intervention to avoid progression or long‐term sequelae.

### Blistering

Blistering may develop when excessive energy is delivered or insufficient epidermal cooling is applied.^26^ It reflects acute thermal injury to the epidermis or dermo‐epidermal junction. Although blisters usually resolve without scarring if properly managed, inappropriate aftercare or secondary infection can prolong healing and increase the risk of post‐inflammatory pigmentary changes. Preventive strategies include meticulous adjustment of fluence and pulse duration to the patient's skin type, as well as the use of effective cooling systems.

### Infectious and Inflammatory Reactions

Disruption of the epidermal barrier predisposes patients to bacterial or viral secondary infection.^27^ Herpes simplex virus reactivation is particularly relevant following ablative procedures. Prophylactic antiviral therapy is recommended for patients with a history of herpes labialis undergoing resurfacing or other high‐risk treatments.^17^, ^18^ To our knowledge, the literature does not provide consistent recommendations regarding dosage or duration for the systematic prophylaxis. In practice, a low‐dose regimen is typically started about 24 hours before the procedure and continued for one to seven days afterward.^28^, ^29^ Strict hygiene measures and appropriate wound care further minimize infection risk.

Acneiform eruptions can appear after certain laser and energy‐based procedures, particularly when occlusive dressings or heavy emollients are used post‐treatment.^30^ Follicular occlusion and local inflammatory responses contribute to this reaction.^30^ Typically, lesions are mild and self‐limiting, but in some cases, topical or systemic therapies may be required. Adjusting post‐procedure skincare and avoiding overly occlusive products are useful preventive measures.

Miliae may form during re‐epithelialization following ablative treatments.^31^ These small, keratin‐filled cysts are benign but may cause cosmetic concern for patients. Extraction with a sterile needle may be performed. Gentle cleansing and non‐comedogenic emollients during the healing phase help reduce their occurrence.

## LATE COMPLICATIONS

Late complications typically last weeks to months after laser and EBD procedures and may become permanent if not recognized and managed promptly.

A thorough understanding of their risk factors, and evidence‐based preventive strategies is essential to optimize patient safety and outcomes.

### Burns

Burns represent a potentially severe complication that can occur when excessive energy parameters are applied, or cooling mechanisms are inadequate. An early warning sign can be the immediate graying of the skin following treatment. Superficial burns may heal with transient pigmentary changes, while deep dermal burns risk permanent scarring or textural changes. Risk is increased in patients with recent tanning, skin of color, or underlying conditions impairing wound healing. Prevention relies on individualized treatment parameter selection and intra‐procedural monitoring. Once a burn occurs, prompt intervention with sufficient cooling, wound care, topical corticosteroids, or antibiotics, if secondary infection is suspected, is necessary to limit long‐term sequelae.

### Pigmentary Alterations

Hypopigmentation and hyperpigmentation are among the most frequent long‐term complications, particularly in patients with skin of color.^32^ Pigmentary alterations often follow excessive fluence or pulse lengths, inadequate cooling, wrong wavelength for skin type, or improper treatment intervals.^33^ Strict photoprotection, cooling, pre‐ and post‐procedure topical agents, and careful parameter adjustment are important strategies for prevention and management.

### Ulceration

**FIGURE 1:**
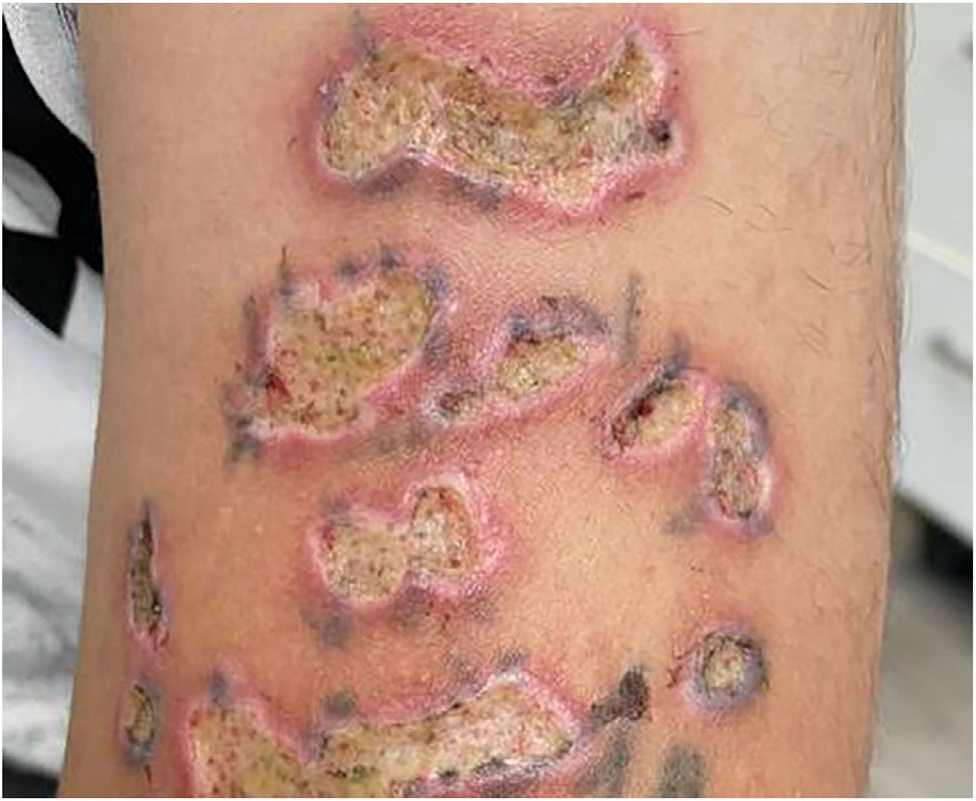
Patient with ulcerations on the arm after laser tattoo removal. Contributing error may be pulse durations longer than thermal relaxation time, high fluences, inappropirate system.

Ulceration is a recognized complication of laser and EBD treatments, typically resulting from excessive energy delivery, longer pulse durations than the thermal relaxation time of the targeted chromophore, or inadequate cooling. It can occur when the epidermis and dermis are damaged beyond the tissue's regenerative capacity, leading to open sores that may increase the risk of secondary infection and delayed healing (Figure 1). Early signs include localized erythema, blistering, or persistent pain at the treatment site. Careful adjustment of device parameters according to skin type and indication, and vigilant post‐treatment care are essential to minimize the risk. Prompt recognition and management, including wound care and infection prevention, are critical to preventing long‐term sequelae such as pigmentary changes and scarring.

### Scarring

**FIGURE 2:**
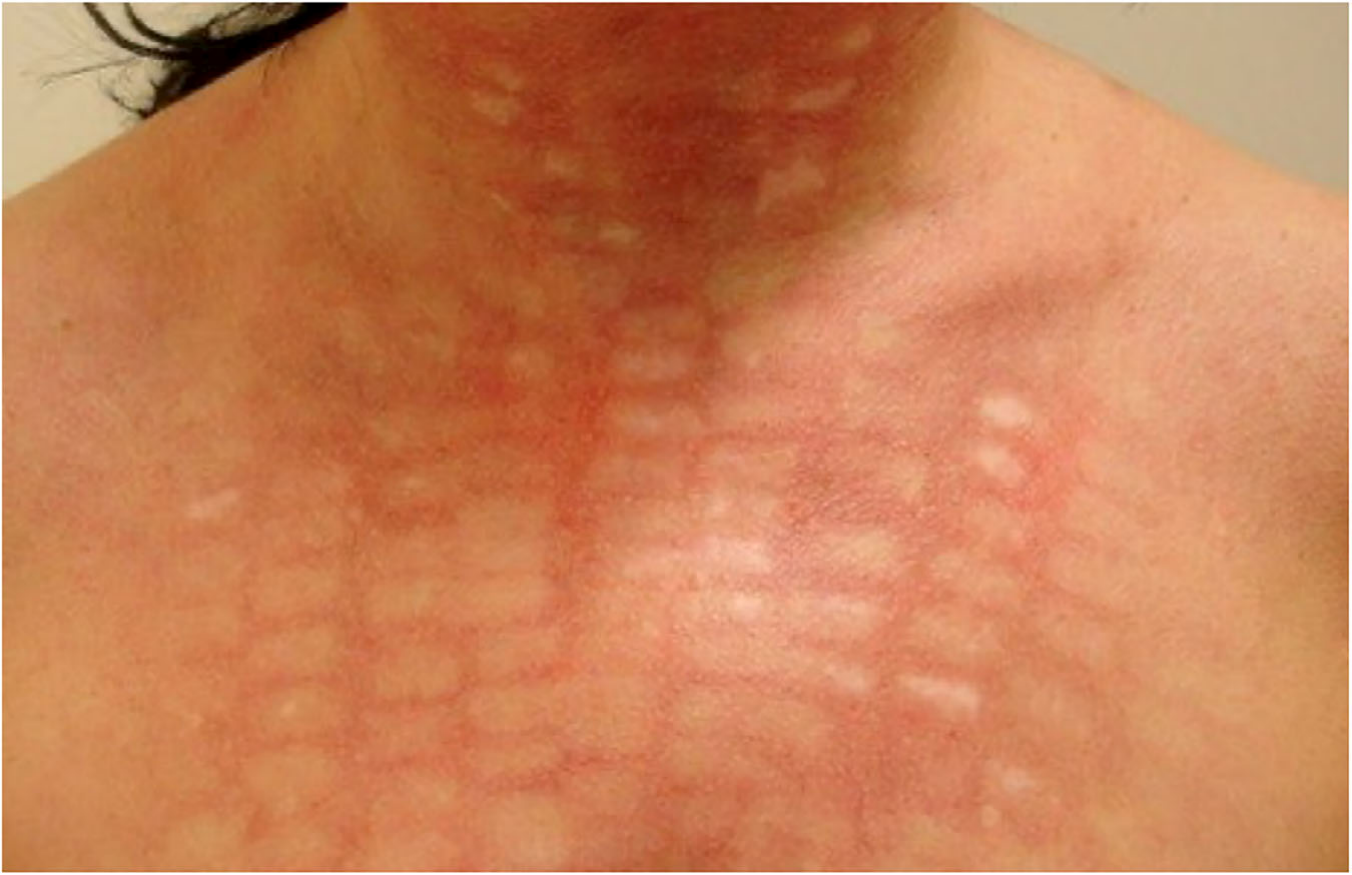
Patient with multiple hypopigmented scars on the decolletage and neck after treatment of erythrosis using intense pulsed light. Contributing error: excessive energy settings.

Scarring constitutes one of the most serious late complications. It can result from deep dermal injury, secondary infections, or improper wound healing following aggressive settings, especially in ablative procedures. Hypertrophic or atrophic scars as well as keloids may occur, causing considerable cosmetic and psychological burden (Figure 2). Prevention relies on careful treatment planning, avoidance of overtreatment, and adequate post‐care. Established scars may require multimodal therapy, including vascular lasers, fractional lasers or corticosteroid injections.

### Paradoxical Darkening

Paradoxical darkening can be observed in tattoo or permanent makeup removal treatments, particularly with certain pigments, e.g. ferric oxide and titanium dioxide. Instead of clearing, the pigment oxidizes and darkens, creating an unfavourable cosmetic outcome.^34^ Prior to treatment test spots may be recommended. Should paradoxical darkening occur, subsequent laser sessions are often effective in gradually improving the outcome.^35^, ^36^

### Paradoxical Hypertrichosis

Paradoxical hair stimulation has been described following laser hair removal, especially when subtherapeutic fluences are applied.^37^ Instead of reducing hair growth, the procedure may stimulate dormant follicles, leading to increased hair density in the treated or adjacent areas.^38^ Adjusting treatment parameters and careful patient selection are key preventive measures.

### Unintended Hair Removal

Laser or EBD treatments not primarily intended for hair removal may nonetheless induce unintended photoepilation if their wavelengths also affect hair follicles. A typical example is the use of long‐pulsed Neodym‐doped Yttrium Aluminum Garnet (Nd:YAG) lasers for vascular treatments, such as rosacea‐associated telangiectasia, in the cheek area of male patients, where hair reduction may occur. Patients should be informed in advance about this potential side effect. Another instance, though generally less pronounced, involves the treatment of permanent makeup in the eyebrow or eyelid margin using Q‐switched Nd:YAG lasers. In these cases as well, patients must be counselled regarding the possible risk of unwanted hair loss prior to the procedure.

### Ocular complications

Ocular complications are rare but potentially serious.

Direct or reflected exposure to laser or intense pulsed light may result in corneal burns, iris deformation, lack of miosis, cataract formation, or retinal injury, depending on the wavelength and energy used.^39^, ^40^, ^41^

Even in non‐periorbital procedures, consistent use of ocular protection is mandatory for both patient and operator. Preventive measures include wavelength‐appropriate protective goggles, intraocular metal eye shields in periocular procedures, and strict adherence to safety protocols.^42^ Plastic eye shields or non‐certified eye protections should be avoided.^42^ Immediate ophthalmologic evaluation is required in the event of suspected ocular injury.

**Table** **1** summarizes the expected side effects as well as immediate and late complications following laser and EBD‐based treatments.

**TABLE 1:** Summary of expected side effects as well as immediate and late complications following treatments laser and energy‐based devices, including their prevention and management.

Category	Subcategory	Details	Aetiology/ Risk Factors	Prevention / Management
**Expected physiological responses**	Erythema	Redness due to thermal/ photomechanical effects	Depends on wavelength, fluence, pulse length, skin sensitivity	Cooling, topical or oral corticosteroids
	Edema	Swelling in treated areas	Often accompanied with erythema, more in loose connective tissue, e.g. periorbital	cooling, head elevation, anti‐inflammatories if needed
	Purpura	Capillary rupture from vascular‐targeting lasers, e.g. in port‐wine birthmarks	Often clinical endpoint in vascular lesions	Self‐limiting; typically resolves 1–2 weeks
	Crust formation	Epidermal disruption from ablative/less‐ablative lasers	Healing process	Gentle cleansing, emollients, infection prevention
**Immediate Complications**	Blistering	Thermal injury to epidermis/ dermo‐epidermal junction	Excessive energy, inadequate cooling	Adjust fluence/ pulse, effective cooling, proper aftercare
	Infection/ Inflammation	Bacterial/ viral infection, herpes reactivation, acneiform eruptions, miliae	Epidermal barrier disruption, occlusive dressings	Prophylactic antivirals, hygiene, gentle cleansing, non‐comedogenic products
**Late Complications**	Burns	Superficial → transient pigment change; deep → scarring	Excess energy, inadequate cooling, darker skin types, recent tanning	Individualized parameters, intra‐procedure monitoring, cooling, prompt wound care
	Pigmentary Alterations	Hyperpigmentation or hypopigmentation	High fluence, pulse length, inadequate cooling, high Fitzpatrick skin types	Strict photoprotection, careful system and parameter selection, topical corticosteroids
	Ulcerations	Destruction of epidermal and dermal layer	High fluence, pulse length, inadequate cooling	Careful planning, constant re‐assessment of parameters during treatment, proper wound care
	Scarring	Hypertrophic, atrophic, keloid	Deep dermal injury, secondary infection, overtreatment	Careful planning, proper post‐care, multimodal therapy for established scars
	Paradoxical Darkening	Darkening of tattoos/ permanent make‐up after laser treatment	Tattoo pigment oxidation (ferric oxide, titanium dioxide)	Test spots, follow‐up laser sessions if occurs
	Paradoxical Hypertrichosis	Increased hair growth after laser hair removal	May result after subtherapeutic fluences	Correct fluence, careful patient selection
	Unwanted hair removal	Epilation effect in non‐photoepilation treatments	Hair growth in treatment area of laser treatment and wavelength which can induce epilation	Patient information and/or avoid areas with hair growth
	Ocular Complications	Corneal burns, iris deformation, lack of miosis, cataract, retinal injury	Direct/ reflected exposure, periocular procedures	Certified protective goggles or intraocular shields, strict safety protocols, ophthalmologic evaluation if injury suspected

Even in non‐periorbital procedures, consistent use of ocular protection is necessary for both patient and operator.

## QUALITY ASSURANCE

Ensuring patient safety and minimizing complications in dermatologic laser and EBD procedures requires a structured approach to quality assurance and prevention.

### Legal Requirements

The NiSV (*Verordnung zum Schutz vor schädlichen Wirkungen nichtionisierender Strahlung bei der Anwendung am Menschen*, which in English translates to: Regulation on protection against harmful effects of non‐ionizing radiation when used on humans), which came into effect in Germany in 2022, establishes legally binding requirements for the safe use of lasers and other non‐ionizing EBDs in aesthetics.^43^

Its primary aim is to enhance patient safety and standardize quality assurance measures for all practitioners. Under NiSV, only individuals with appropriate medical training may operate high‐risk devices such as lasers and EBDs.^43^ Physicians are required to complete certified training, including theoretical and practical modules, to demonstrate competence in device operation, patient selection, and complication management.^43^ Non‐physicians are restricted in the use of these devices and must adhere to strict supervision and scope regulations.^43^ These requirements ensure that only adequately trained personnel perform potentially high‐risk procedures, aligning with best‐practice principles in dermatologic laser therapy. Furthermore, according to NiSV, only licensed physicians may perform treatments that include damage of the epidermal barrier, such as tattoo removal, ablative laser procedures, and other comparable therapies, provided they are dermatologists, plastic surgeons, or have obtained the specific NiSV certification for such treatments.^43^

The regulation also mandates detailed documentation of all procedures, including patient consent, treatment parameters, and any adverse events.^43^ Structured record‐keeping supports continuous quality improvement and enables the tracking of complications for both internal audit and external review. Additionally, NiSV requires regular device inspection, maintenance, and calibration to ensure safe and consistent energy delivery. By enforcing standardized safety checks and adherence to manufacturer guidelines, the regulation reduces the risk of equipment‐related complications. Overall, NiSV reinforces a culture of quality assurance in laser and EBD practice.

By combining mandatory physician training, thorough documentation, device safety measures, and legal oversight, it provides a robust framework for preventing complications and maintaining high standards of patient care.

### Comprehensive Training and Competence

A critical prerequisite for safe laser application is proper physician training. Formal programs such as the *Master of Science in Differentiated Aesthetic Laser and Plasma Medicine (DALM)* provide structured, evidence‐based curricula that ensure physicians acquire in‐depth knowledge of laser physics, device‐specific parameters, indications, contraindications, and complication management.^44^, ^45^

Training should emphasize hands‐on experience under expert supervision. Importantly, the use of medical lasers and EBDs should remain firmly within the scope of qualified physicians.

### Systematic Monitoring and Complication Registries

An additional cornerstone of quality assurance is the systematic monitoring of adverse events. Establishing and maintaining registries for laser‐ and EBD‐related complications enables continuous learning and facilitates the identification of risk patterns across devices and treatment settings. Such registries, like the complication registry of the German Society of Laser Dermatology (Deutsche Dermatologische Lasergesellschaft, DDL) allow physicians to assess their outcomes against national or international data, thereby supporting continuous improvement and enhancing patient safety. Furthermore, registry data can inform future guideline development and training curricula, creating a feedback loop between clinical practice and medical education.

## CONCLUSIONS

Lasers and EBDs represent a cornerstone of contemporary dermatology, offering a broad spectrum of therapeutic modalities for a variety of clinical and aesthetic indications. However, their safe and effective use requires a deep understanding of the associated risk factors, expected physiological responses, and potential complications. While most adverse effects are transient and self‐limited, long‐term sequelae such as pigmentary alterations, scarring, or ocular injury highlight the importance of prevention, early recognition, and evidence‐based management. Legal frameworks such as the NiSV, structured physician training, rigorous documentation, and complication registries play a pivotal role in standardizing practice and ensuring patient safety. The integration of advanced technology with quality assurance and professional expertise is essential to minimize risks, optimize outcomes, and maintain high standards of dermatologic care. Future developments should also consider supervisory authorities to further enhance safety and accountability.

## CONFLICT OF INTEREST STATEMENT

LN has received lecture fees from Cynosure Lutronic®. SH is the scientific director of the postgraduate program Master of Science in Differentiated Aesthetic Laser and Plasma Medicine (DALM). NS is the president of the Deutsche Dermatologische Lasergesellschaft (DDL). WK and SWS have none to be declared.

Open access funding enabled and organized by Projekt DEAL.

## [CME Questions – Lernerfolgskontrolle]

1. Was beschreibt die Theorie der selektiven Photothermolyse?
  - Die kontinuierliche Abgabe von Laserenergie unabhängig von Absorptionscharakteristika der Haut.
  - Die gezielte Zerstörung spezifischer Zielstrukturen (Chromophore) im Gewebe durch selektive Absorption von Laserenergie unter Schonung des umliegenden Gewebes.
  - Die unspezifische Erwärmung aller Gewebeschichten durch breitbandiges Licht, die zu einer globalen Thermoschädigung führt.
  - Die Erhöhung der Kollagenproduktion durch subtherapeutische Wärmeeinwirkung ohne direkte Gewebsdestruktion.
  - Die vollständige Abtragung der Epidermis und Dermis mit anschließender Reepithelisierung.
2. Welche Aussage ist richtig?
  - Die Parameter der Gerätehersteller können für jeden Patienten einfach übernommen werden.
  - Höhere Fluenzwerte sind grundsätzlich sicherer, da sie schneller wirken.
  - Die Parameter sollten je nach Indikation und Patienten‐Charakteristika angepasst werden.
  - Patientensicherheit hängt ausschließlich von der Gerätequalität ab, nicht vom Anwender.
  - Eine Anpassung der Pulsdauer ist nicht notwendig, solange das Gerät CE‐zertifiziert ist.
3. Was sind nicht zu erwartende Reaktionen der Haut nach einer Behandlung mit einem Laser oder Energie‐basierten System?
  - Erythem
  - Schwellung
  - Erhöhung der Leberwerte
  - Purpura
  - Krusten
4. Welche Aussage stimmt?
  - Man kann einfach über infizierte Areale lasern, da die Laserenergie die Keime abtötet.
  - In bestimmten Fällen ist eine präventive Herpes‐Therapie empfohlen.
  - Aktive bakterielle, virale oder mykotische Infektionen an der zu behandelnden Stelle stellen keine Kontraindikation für eine Laserbehandlung dar.
  - Hygienestandards und sorgfältige Wundpflege sind für die Infektionsprophylaxe nicht notwendig.
  - Nach ablativen Laserbehandlungen besteht kein Risiko einer Infektion, da die Hautbarriere intakt bleibt.
5. Langzeitkomplikationen nach Laser‐ und EBD‐Behandlungen können …
  - immer vollständig ausgeschlossen werden.
  - unter anderem Narbenbildung und Keloide umfassen.
  - ausschließlich bei Patienten mit dunklem Hauttyp nach Fitzpatrick auftreten.
  - durch routinemäßige Antibiotikagabe sicher verhindert werden.
  - ohne klinische Bedeutung sein und bedürfen keiner weiteren Behandlung.
6. Welche Aussage ist falsch?
  - Während einer Laserbehandlung müssen sowohl der Patient als auch der Behandler einen suffizienten Augenschutz tragen.
  - Im Rahmen von Laserbehandlungen können schwarze Augenschalen aus Kunststoff zum Schutz der Augen genutzt werden.
  - Der Behandler kann auf Augenschutz verzichten, wenn er den Laser nicht direkt in Richtung seiner Augen richtet.
  - Der Augenschutz muss für die jeweilige Laserwellenlänge geeignet sein.
  - Bei periokulären Behandlungen können Metall‐Augenschalen zum Einsatz kommen
7. Was reguliert die Nicht‐Ionisierende Strahlquellen‐Verordnung (NiSV)?
  - Die Anwendung von Laser‐ und anderen nichtionisierenden Strahlungsquellen zu kosmetischen Zwecken.
  - Den Arbeitsschutz beim Umgang mit ionisierender Strahlung (z. B. Röntgen, Computer‐Tomographie).
  - Die Herstellung und Zulassung von medizinischen Geräten mit Laser‐ oder Ultraschalltechnologie.
  - Die Strahlendosisgrenzwerte für radioaktive Substanzen in der Nuklearmedizin.
  - Die Vergütung von Laser‐ und Strahlentherapien durch gesetzliche Krankenkassen.
8. Wer darf laut der Nicht‐Ionisierenden Strahlquellen‐Verordnung (NiSV) eine Behandlung mit Lasersystemen und Energie‐basierten Geräten durchführen?
  - Jede kosmetische Fachkraft nach einer einwöchigen Geräteeinweisung durch den Hersteller.
  - Heilpraktikerinnen und Heilpraktiker ohne zusätzliche Qualifikation.
  - Alle Personen, die eine NiSV‐Fachkundeprüfung im Bereich Haut absolviert haben, unabhängig von der Indikation.
  - Nur approbierte Ärztinnen und Ärzte, wenn es sich um Therapien handelt, die die Epidermisbarriere schädigen (z. B. Tattooentfernung, ablative Therapien, etc.), sofern sie Dermatologen oder plastische Chirurgen sind oder die entsprechende NiSV‐Fachkunde erworben haben.
  - Kosmetikerinnen und Kosmetiker, sofern sie eine zweijährige Berufserfahrung nachweisen können.
9. Was beinhaltet die Nicht‐Ionisierende Strahlquellen‐Verordnung nicht?
  - Regelungen zur Anwendung von Lasern, intensiven gepulsten Lichtquellen, Ultraschall und Hochfrequenzgeräten zu nichtmedizinischen Zwecken.
  - Anforderungen an die Fachkunde und regelmäßige Fortbildung des anwendenden Personals.
  - Vorgaben zur Aufklärung, Dokumentation und zum Schutz der behandelten Personen.
  - Bestimmungen zur Herstellung und zum Inverkehrbringen von medizinischen Geräten.
  - Schutzmaßnahmen vor gesundheitlichen Risiken bei kosmetischen Laser‐ und Energieanwendungen.
10. Welchen Nutzen hat der DALM‐Studiengang (Master of Science in Differentiated Aesthetic Laser and Medicine)?
  - Er vermittelt eine strukturierte, evidenzbasierte Ausbildung in Laser‐ und energiebasierten Verfahren, einschließlich Indikationen, Kontraindikationen und Komplikationsmanagement.
  - Er berechtigt automatisch zur Anwendung aller kosmetischen Verfahren ohne zusätzliche Fachkunde.
  - Er ersetzt die gesetzlich vorgeschriebene NiSV‐Fachkunde vollständig.
  - Er dient ausschließlich der Geräteeinweisung durch Herstellerfirmen.
  - Er ist ausschließlich für Kosmetiker konzipiert.

Liebe Leserinnen und Leser, der Einsendeschluss an die DDA für diese Ausgabe ist der 29. Mai 2026.

Die richtige Lösung zum Thema Sklerosierende Erkrankungen der Haut in Heft 10/2025 ist: 1a, 2e, 3c, 4b, 5e, 6d, 7b, 8c, 9a, 10c

Bitte verwenden Sie für Ihre Einsendung das aktuelle Formblatt auf der folgenden Seite oder aber geben Sie Ihre Lösung online unter http://jddg.akademie-dda.de ein.

## References

[1] Raulin C , Karsai S . Laser and IPL technology in dermatology and aesthetic medicine. 2011;10:3‐40.

[2] Anderson RR . Lasers in dermatology—a critical update. J Dermatol. 2000;27(11):700‐705. 11138535 10.1111/j.1346-8138.2000.tb02262.x

[3] Anderson RR , Parrish JA . Selective photothermolysis: precise microsurgery by selective absorption of pulsed radiation. Science. 1983;220(4596):524‐527. 6836297 10.1126/science.6836297

[4] Stuart Nelson J , Milner TE , Svaasand LO , Kimel S . Laser pulse duration must match the estimated thermal relaxation time for successful photothermolysis of blood vessels. Lasers Med Sci. 1995;10(1):9‐12.

[5] Zenzie H , Altshuler G , Smirnov M , Anderson R . Evaluation of cooling methods for laser dermatology. Lasers Surg Med. 2000;26(2):130‐144. 10685086 10.1002/(sici)1096-9101(2000)26:2<130::aid-lsm4>3.0.co;2-j

[6] Bodendorf MO , Grunewald S , Simon JC , Paasch U . Efficacy and cosmetic results of contact gel cooling of the skin during non‐ablative laser procedures. J Dtsch Dermatol Ges. 2008;6(8):647‐652. 18201219 10.1111/j.1610-0387.2008.06610.x

[7] Raulin C , Greve B , Hammes S . Cold air in laser therapy: first experiences with a new cooling system. Lasers Surg Med. 2000;27(5):404‐410 11126434 10.1002/1096-9101(2000)27:5<404::AID-LSM1001>3.0.CO;2-S

[8] Lack EB , Rachel JD , D'Andrea L , Corres J . Relationship of energy settings and impedance in different anatomic areas using a radiofrequency device. Dermatol Surg. 2005;31(12):1668‐1670. 16336885 10.2310/6350.2005.31306

[9] Willey A , Anderson RR , Azpiazu JL , et al. Complications of laser dermatologic surgery. Lasers Surg Med. 2006;38(1):1‐15. 16444692 10.1002/lsm.20286

[10] Hammes S , Karsai S , Metelmann HR , et al. Treatment errors resulting from use of lasers and IPL by medical laypersons: results of a nationwide survey. J Dtsch Dermatol Ges. 2013;11(2):149‐156. 23194381 10.1111/j.1610-0387.2012.08042.x

[11] Soares I , Amaral IP , Correia MP , et al. Complications of dermatologic lasers in high Fitzpatrick phototypes and management: an updated narrative review. Lasers Med Sci. 2024;39(1):149. 38834924 10.1007/s10103-024-04100-4

[12] Shah S , Alster TS . Laser treatment of dark skin: an updated review. Am J Clin Dermatol. 2010;11(6):389‐397. 20866114 10.2165/11538940-000000000-00000

[13] jr Battle EF , jr Soden CE , editors. The use of lasers in darker skin types. Semin Cutan Med Surg; 2009: WB Saunders. 10.1016/j.sder.2009.04.003 19608064

[14] Kalka K , Merk H , Mukhtar H . Photodynamic therapy in dermatology. J Am Acad Dermatol. 2000;42(3):389‐413. 10688709 10.1016/s0190-9622(00)90209-3

[15] Association BML . Drugs and Lasers/IPLs. https://bmla.co.uk/drugs‐and‐laser‐ipls/2018 [Last accessed December 10, 2025].

[16] Kerstein RL , Lister T , Cole R . Laser therapy and photosensitive medication: a review of the evidence. Lasers Med Sci. 2014;29(4):1449‐1452. 24590242 10.1007/s10103-014-1553-0

[17] Alster TS , Nanni CA . Famciclovir prophylaxis of herpes simplex virus reactivation after laser skin resurfacing. Dermatol Surg. 1999;25(3):242‐246. 10193975 10.1046/j.1524-4725.1999.08197.x

[18] Beeson WH , Rachel JD . Valacyclovir prophylaxis for herpes simplex virus infection or infection recurrence following laser skin resurfacing. Dermatol Surg. 2002;28(4):331‐336. 11966791 10.1046/j.1524-4725.2002.01155.x

[19] Haedersdal M , Bech‐Thomsen N , Poulsen T , Wulf HC . Ultraviolet exposure influences laser‐induced wounds, scars, and hyperpigmentation: a murine study. Plast Reconstr Surg. 1998;101(5):1315‐1322. 9529218 10.1097/00006534-199804050-00024

[20] Duke D , Grevelink JM . Care before and after laser skin resurfacing. A survey and review of the literature. Dermatol Surg. 1998;24(2):201‐206. 9491114 10.1111/j.1524-4725.1998.tb04138.x

[21] Gold M , Andriessen A , Cohen JL , et al. Pre‐/postprocedure measures for laser/energy treatments: A survey. J Cosmet Dermatol. 2020;19(2):289‐295. 31840388 10.1111/jocd.13259

[22] Nanni CA , Alster TS . Complications of cutaneous laser surgery. A review. Dermatol Surg. 1998;24(2):209‐219. 9491115 10.1111/j.1524-4725.1998.tb04139.x

[23] Adamič M , Pavlović M , Troilius Rubin A , et al. Guidelines of care for vascular lasers and intense pulse light sources from the European Society for Laser Dermatology. J Eur Acad Dermatol Venereol. 2015;29(9):1661‐1678. 25931003 10.1111/jdv.13177

[24] Alam M , Dover JS , Arndt KA . Treatment of facial telangiectasia with variable‐pulse high‐fluence pulsed‐dye laser: comparison of efficacy with fluences immediately above and below the purpura threshold. Dermatol Surg. 2003;29(7):681‐684. 12828690 10.1046/j.1524-4725.2003.29181.x

[25] Alexiades‐Armenakas MR , Dover JS , Arndt KA . The spectrum of laser skin resurfacing: nonablative, fractional, and ablative laser resurfacing. J Am Acad Dermatol. 2008;58(5):719‐737. 18423256 10.1016/j.jaad.2008.01.003

[26] Zelickson Z , Schram S , Zelickson B . Complications in cosmetic laser surgery: a review of 494 Food and Drug Administration manufacturer and user facility device experience reports. Dermatol Surg. 2014;40(4):378‐382. 24826394 10.1111/dsu.12461

[27] Chiller K , Selkin BA , Murakawa GJ , editors. Skin microflora and bacterial infections of the skin. J Investig Dermatol Symp Proc; 2001: Elsevier. 10.1046/j.0022-202x.2001.00043.x 11924823

[28] Alster TS , Nanni CA . Famciclovir prophylaxis of herpes simplex virus reactivation after laser skin resurfacing. Dermatol Surg. 1999;25(3):242‐246. 10193975 10.1046/j.1524-4725.1999.08197.x

[29] Beeson WH , Rachel JD . Valacyclovir prophylaxis for herpes simplex virus infection or infection recurrence following laser skin resurfacing. Dermatol Surg. 2002;28(4):331‐336. 11966791 10.1046/j.1524-4725.2002.01155.x

[30] Mirza HN , Mirza FN , Khatri KA . Outcomes and adverse effects of ablative vs nonablative lasers for skin resurfacing: a systematic review of 1093 patients. Dermatol Ther. 2021;34(1):e14432. 33084193 10.1111/dth.14432

[31] Kaur J , Sharma S , Kaur T , Bassi R . Complications of fractional ablative carbon dioxide laser in various aesthetic procedures: a retrospective study. Int J Res Dermatol. 2019;5(4):664‐667.

[32] Kim YJ , Lee HS , Son SW , et al. Analysis of hyperpigmentation and hypopigmentation after Er: YAG laser skin resurfacing. Lasers Surg Med. 2005;36(1):47‐51. 15662626 10.1002/lsm.20120

[33] Graber EM , Tanzi EL , Alster TS . Side effects and complications of fractional laser photothermolysis: experience with 961 treatments. Dermatol Surg. 2008;34(3):301‐307. 18190541 10.1111/j.1524-4725.2007.34062.x

[34] Anderson RR , Geronemus R , Kilmer SL , et al. Cosmetic tattoo ink darkening: a complication of Q‐switched and pulsed‐laser treatment. Arch Dermatol. 1993;129(8):1010‐1014. 8352605 10.1001/archderm.129.8.1010

[35] Bae YSC , Alabdulrazzaq H , Brauer J , Geronemus R . Successful treatment of paradoxical darkening. Lasers Surg Med. 2016;48(5):471‐473. 26833886 10.1002/lsm.22482

[36] Kang S , Park S , Park J , et al. Paradoxical darkening following picosecond laser and successful treatment. Clin Exp Dermatol. 2021;46(6):1128‐1129. 33774841 10.1111/ced.14661

[37] Desai S , Mahmoud BH , Bhatia AC , Hamzavi IH . Paradoxical hypertrichosis after laser therapy: a review. Dermatol Surg. 2010;36(3):291‐298. 20100274 10.1111/j.1524-4725.2009.01433.x

[38] Town G , Bjerring P . Is paradoxical hair growth caused by low‐level radiant exposure by home‐use laser and intense pulsed light devices? J Cosmet Laser Ther. 2016;18(6):355‐362. 26983796 10.3109/14764172.2016.1157373

[39] Huang A , Phillips A , Adar T , Hui A . Ocular injury in cosmetic laser treatments of the face. J Clin Aesthet Dermatol. 2018;11(2):15. PMC5843357 29552271

[40] Juhasz M , Zachary C , Cohen JL . Ocular complications after laser or light‐based therapy—dangers dermatologists should know. Dermatol Surg. 2021;47(5):624‐629. 33731574 10.1097/DSS.0000000000002974

[41] Flegel L , Kherani F , Richer V . Review of eye injuries associated with dermatologic laser treatment. Dermatol Surg. 2022;48(5):545‐550. 35333214 10.1097/DSS.0000000000003427

[42] Nguyen L , Seeber N , Schneider SW , Herberger K . Thermal eye injuries from dermatologic laser treatments—an experimental study. Lasers Med Sci. 2023;38(1):110. 37086295 10.1007/s10103-023-03769-3 PMC10122618

[43] Verordnung zum Schutz vor schädlichen Wirkungen nichtionisierender Strahlung bei der Anwendung am Menschen (NiSV). BGBl. I Nr. 25/2020. 2020. https://www.gesetze‐im‐internet.de/nisv/BJNR218700018.html?utm_source=chatgpt.com. [Last accessed September 28, 2025].

[44] Metelmann H‐R , Hammes S , Hartwig K , et al. Safe and Effective Plasma Treatment by Structured Education. Comprehensive Clinical Plasma Medicine: Cold Physical Plasma for Medical Application: Springer; 2018;467‐472.

[45] Metelmann H‐R , Seebauer C , Hammes S . Postgraduate Education to Assure Quality Standards of Photonic Treatment. Energy for the Skin: Effects and Side‐Effects of Lasers, Flash Lamps and Other Sources of Energy. In: Energy for the Skin. Ed: Kautz G. Springer; 2022;31‐36.

